# The Small GTPase Rab5c Exerts Bi-Function in Singapore Grouper Iridovirus Infections and Cellular Responses in the Grouper, *Epinephelus coioides*

**DOI:** 10.3389/fimmu.2020.02133

**Published:** 2020-09-02

**Authors:** Liqun Wang, Chen Li, Xinyue Zhang, Min Yang, Shina Wei, Youhua Huang, Qiwei Qin, Shaowen Wang

**Affiliations:** ^1^Joint Laboratory of Guangdong Province and Hong Kong Region on Marine Bioresource Conservation and Exploitation, College of Marine Sciences, South China Agricultural University, Guangzhou, China; ^2^College of Fisheries, Henan Normal University, Xinxiang, China; ^3^Guangdong Laboratory for Lingnan Modern Agriculture, Guangzhou, China; ^4^Laboratory for Marine Biology and Biotechnology, Qingdao National Laboratory for Marine Science and Technology, Qingdao, China

**Keywords:** grouper, Rab5c, SGIV, viral infection, immune responses

## Abstract

The small GTPase Rab5 is one of the master regulators of vesicular trafficking that participates in early stages of the endocytic pathway, such as endocytosis and endosome maturation. Three Rab5 isoforms (a, b, and c) share high sequence identity, and exhibit complex functions. However, the role of Rab5c in virus infection and cellular immune responses remains poorly understood. In this study, based on the established virus-cell infection model, Singapore grouper iridovirus (SGIV)-infected grouper spleen (GS) cells, we investigated the role of Rab5c in virus infection and host immune responses. Rab5c was cloned from the orange-spotted grouper, *Epinephelus coioides*, and termed EcRab5c. EcRab5c encoded a 220-amino-acid polypeptide, showing 99% and 91% identity to *Anabas testudineus*, and *Homo sapiens*, respectively. Confocal imaging showed that EcRab5c localized as punctate structures in the cytoplasm. However, a constitutively active (CA) EcRab5c mutant led to enlarged vesicles, while a dominant negative (DN) EcRab5c mutant reduced vesicle structures. EcRab5c expression levels were significantly increased after SGIV infection. EcRab5c knockdown, or CA/DN EcRab5c overexpression significantly inhibited SGIV infection. Using single-particle imaging analysis, we further observed that EcRab5c disruption impaired crucial events at the early stage of SGIV infection, including virus binding, entry, and transport from early to late endosomes, at the single virus level. Furthermore, it is the first time to investigate that EcRab5c is required in autophagy. Equally, EcRab5c positively regulated interferon-related factors and pro-inflammatory cytokines. In summary, these data showed that EcRab5c exerted a bi-functional role on iridovirus infection and host immunity in fish, which furthers our understanding of virus and host immune interactions.

## Introduction

The small GTPase Rab5, is a marker of early endosomes (EE), is one of the “housekeeping” Rab, and cycles between GTP- and GDP-bound conformations (1). Rab5-GTP localizes to the endosomal membrane, and Rab5-GDP is dispersed in the cytosol, and is associated with the Rab-GDP dissociation inhibitor (GDI) (2). Rab5 effectors, such as early-endosome antigen 1 (EEA1) and rabankyrin-5, bind not to Rab5-GDP, but to Rab5-GTP; thus a key determinant of Rab5 function is the duration of the GTP-bound state (3–5). In addition, approximately 20 cytosolic proteins specifically interact with active Rab5, suggesting a complex regulatory network (3). The primary Rab5 functions are involved in endocytic trafficking. Firstly, Rab5 is an important regulator of clathrin-mediated endocytosis (CME), controlling clathrin-coated vesicles entering cells and fusing with EE (6). For example, transferrin endocytosis is enhanced by Rab5 overexpression, and is impaired by the overexpression of a Rab5 mutant (7). Secondly, Rab5 participates in caveolar-mediated endocytosis, by targeting caveolar vesicles to the EE, and regulating traffic between caveolae and caveosomes (8). Thirdly, Rab5 is involved in actin-rich membrane ruffling and micropinosomes (9, 10). Finally, Rab5 is an important regulator of endosome maturation, fusion and trafficking (11). The Rab5-to-Rab7 switch machinery executes conversion from the EE to late endosome (LE). In addition, Rab5 is also associated with immune responses, such as phagocytosis and autophagy (12).

It has been demonstrated that numerous viruses, such as influenza virus (IV), Semliki Forest virus, adenovirus (AdV), vesicular stomatitis virus, and Japanese encephalitis virus, require Rab5 for survival (13–15). Rab5 participates in the RNA replication of hepatitis C virus (HCV) by interacting with NS4B, and facilitates the formation of replication complexes (16, 17). Rab5 is also necessary for the envelopment of herpes simplex virus 1 (HSV1) (18). Other than viruses, some bacteria have also evolved mechanisms to survive within specialized immature phagosomes, by manipulating Rab5 functions. For instance, *Mycobacterium tuberculosis* replicate in phagosomes that are blocked in the Rab5-positive stage (19). *Leishmania donovani* survives in macrophages, by up-regulating Rab5a expression (20).

Iridoviruses are large double-stranded DNA viruses, composed of a spherical deoxyribonucleoprotein core and a lipid membrane (21). They infect a broad range of hosts, such as valuable invertebrates and poikilothermic vertebrates, including crustaceans, fish, amphibians, and reptiles, causing serious losses to aquaculture and biodiversity (22, 23). The Singapore grouper iridovirus (SGIV) belongs to the genus *Ranavirus*, family *Iridoviridae*, and causes high mortality and production losses in grouper aquaculture (22). Our previous studies demonstrated that SGIV enters host cells through CME and micropinocytosis pathways, depending on low pH for successful infection (24). In addition, EcRab7 overexpression promotes SGIV transcription (25). Therefore, it is speculated that Rab5 also plays roles in the SGIV life cycle. The three Rab5 isoforms (Rab5a, -b, and -c) share more than 90% sequence identity, yet are encoded by different genes (26). It is reported that Rab5 isoforms show similar localization and capabilities in regulating endocytosis, yet some functional differences are evident (7). Many studies have focused on Rab5a, however, few have investigated Rab5c, especially on the impact of virus infection and cellular immune responses.

In this study, EcRab5c, derived from grouper *Epinephelus coioides*, was cloned and investigated. Notably, EcRab5 plays important and diverse roles in both SGIV infection and cellular innate immunity; EcRab5c is crucial to the SGIV life cycle; including SGIV attachment, entry and transport, whereas in addition, EcRab5c positively regulates cellular innate immunity, including autophagy, IFN, and inflammatory responses. These data suggest a complex role for EcRab5c in virus infection and cellular immunity, and provides new insights on interactions between virus and host cells, potentially contributing to the development of new antiviral strategies.

## Materials and Methods

### Fish, Cells and Viruses

A grouper spleen (GS) cell line was established in our laboratory, and maintained at 28° C in Leibovitz’s L-15 medium, containing 10% fetal bovine serum (Gibco, United States) (27). SGIV stocks were isolated from diseased grouper (*Epinephelus tauvina*), as described previously (22).

### Reagents

Peroxidase-conjugated affinipure goat anti-rabbit IgG was purchased from Proteintech, United States. Rabbit monoclonal anti-LC3 antibody, anti-β-tubulin and anti-Rab5c were purchased from Abcam, United States. Hoechst 33342 and Rap were purchased from Sigma-Aldrich. The fluorescent label, Alexa Fluor 647 and 4% paraformaldehyde were purchased from Invitrogen. The lipophilic dye, DiO was purchased from Biotium, United States. The SGIV polyclonal anti-MCP antibody was prepared in our laboratory. The labeled si-EcRab5c sequence is listed in Supplementary Table 1, and was synthesized by GenePharma, China.

### Virus Purification and Fluorescence Labeling

Purified and fluorescence-labeled SGIV particles are described below. GS cell monolayers were infected with SGIV [multiplicity of infection (MOI) = 0.1]. SGIV particles were collected by repeated freeze-thaw cycles, centrifuged at 12,000 × *g* (Beckman Allegra X-15R Centrifuge) for 30 min at 4° C, after which the supernatant was ultracentrifuged at 200,000 *g* (Beckman 70 Ti rotor) for 1 h at 4° C. Subsequently, the pellet was resuspended in TN buffer (50 mM Tris–HCl, 150 mM NaCl, and pH 7.5) and layered onto a sucrose gradient (30–60%, wt/vol) for another ultracentrifugation step at 150,000 g (Beckman SW 40 rotor) for 1 h at 4° C. The purified viral band was collected in TN buffer, ultracentrifuged at 100,000 × *g* for 1 h at 4° C, resuspended in TN buffer, and then stored at −80° C until required.

Singapore grouper iridovirus particles were incubated with Alexa Fluor 647 in phosphate-buffered saline (PBS; pH 7.4) at room temperature for 2 h, with slight vortexing. Then, unincorporated dye was removed by three high-speed centrifugation steps at 14,000 × *g* (Beckman Micrifuge 20R Centrifuge) for 1 h at 4° C. The labeled virus was visualized under a transmission electron microscope and preserved at 4° C.

### Identification and Bioinformatics Analysis of EcRab5c

According to the expressed sequence tag (EST) of the grouper transcriptome, the ORF of EcRab5c was amplified by specific primers (Supplementary Table 1) using PCR. Analysis of the EcRab5c putative amino acid sequence was performed using the BLAST program in NCBI^1^. The domain structure of EcRab5c was predicted by SMART^2^. Multiple sequence alignments were examined using Clustalx 1.83^3^, and results were edited by the GeneDoc software. EcRab5c phylogenetic analyses were performed by MEGA 6.0 software.

### EcRab5c Expression Patterns

To further verify the effects of viral infection on EcRab5c expression, GS cells were infected with SGIV (MOI = 1) and harvested at 18, 24, and 36 hpi for Western blotting. β-tubulin was used as an internal control.

### RNA Extraction and qRT-PCR

According to manufacturer’s instructions, total RNA was extracted using the SV Total RNA Isolation System (Promega, United States) and examined by electrophoresis. A cDNA synthesis kit, ReverTraAce (TOYOBO, Japan), was used to reversibly transcribe RNA into cDNA. qRT-PCR was performed using a Roche 480 Real Time Detection System (Roche, Basel, Switzerland), and was carried out according to manufacturer’s protocols (TOYOBO). β-actin was used as an internal control (Supplementary Table 1). The other primers listed in this table were also used for qRT-PCR, and include the host IFN signaling molecules (IFN, IRF7, ISG15, MyD88, IFP35, TRAF6, and MDA5), pro-inflammatory factors (TNF-α, IL-6, and IL-1β) and SGIV genes (ICP18, VP49, and MCP). Each assay was performed under the following cycling conditions: 94° C for 5 min, followed by 45 cycles of 5 s at 94° C, 10 s at 60° C, and 15 s at 72° C. The data were calculated as fold changes based on the normalization of targeted gene transcription levels with β-actin, and presented in terms of relative mRNA transcription levels [means ± standard error of the mean (SEM)]. Each assay was performed in triplicate. Statistical significances were determined using Student’s *t*-tests (^∗^*p* < 0.05).

### Plasmid Construction

To determine EcRab5c function *in vitro*, the full-length EcRab5c was cloned into pEGFP-C1 and pcDNA3.1-Flag (Invitrogen, United States) plasmids, using PCR primers (Supplementary Table 1), and verified by sequencing. The EcRab5c glutamine codon (Q) at position 80, and serine codon (S) at position 35 were mutated to leucine (L) and asparagine (N), respectively, using the Fast Mutagenesis Kit V2 (Vazyme, China) to create the mutant plasmids, pEGFP-EcRab5c-Q80L (CA EcRab5c) and pEGFP-EcRab5c-S35N (DN EcRab5c). The pEGFP-EcRab7 and pEGFP-LC3 plasmids were maintained in our laboratory.

### Western Blotting

Grouper spleen cells were collected and lysed in Pierce IP Lysis Buffer (Thermo Scientific, United States). Proteins were separated by 12% SDS-PAGE and transferred onto Immobilon-polyvinylidene difluoride membranes (Millipore, Temecula, CA, United States). Blots were incubated in 5% skim milk for 2 h, and were incubated with indicated primary antibodies: anti-Rab5c (1:500 dilution), anti-β-tubulin (1:2,000 dilution), anti-SGIV major capsid protein (MCP; 1:1,000 dilution), and anti-LC3 (1:1,000 dilution). After washing three times in PBS Tween membranes were incubated with peroxidase-conjugated affinipure goat anti-rabbit IgG (1:5,000 dilution). Immuno-reactive proteins were visualized using an enhanced HRP-DAB Chromogenic Substrate Kit (Tiangen, China) and SuperSignal (R) West Femto Trial Kit (Thermo Scientific, United States).

### Cell Transfections

Grouper spleen cells grown to 50–70% confluence were transiently transfected with plasmids or si-RNA, using Lipofectamine 2000 (Invitrogen) according to manufacturer’s instructions. For plasmids, 0.8 μg DNA and 2 μl Lipofectamine 2000 were diluted in 100 μl serum-free medium, with OptiMEM and L-15 medium (1:1). For si-RNA transfections, 160 nM si-RNA and 2 μl Lipofectamine 2000 were diluted in 100 μl serum-free medium containing OptiMEM and L-15 medium (1:1). Then, mixtures were added to cells and incubated at 28° C. After 6 h, serum-free medium replaced the serum-containing medium, for further culture.

### Virus Infection Assay

To evaluate the effects of EcRab5c on virus infection, GS cells were cultured in 24-well plates previously transfected with an si-control and si-EcRab5c. Cells were then incubated with SGIV (MOI = 1). At 24 hpi, cytopathic effects (CPE) were observed under an inverted light microscopy. Cells were then harvested for RNA extraction and Western blotting.

### SGIV Attachment and Internalization Assays

Viral attachment and internalization assays were performed by measuring SGIV particles at cell boundaries and in the cytoplasm, respectively. In brief, GS cells transfected with si-control or si-EcRab5c were infected with Alex-Fluor 647 labeled SGIV (MOI = 10) for 1 h at 4° C. For the attachment assay, cells were immediately fixed after SGIV incubation at 4° C, and observed using confocal microscopy. For the internalization assay, cells were rapidly transferred to 28° C to initiate infection, fixed at 1 hpi, and then observed by confocal microscopy. Approximately 100 cells were randomly analyzed.

### Confocal Microscopy and Single-Particle Imaging Assay

Fluorescent images were observed on a ZEISS LSM 7 DUO confocal microscope. A 488-nm Ar-Kr laser was used to excite EGFP and DiO signals, and a 500–550 nm bandpass filter was used for emission. A 533 nm laser was used to excite the Cy3 signal, and a 540–590 nm bandpass filter was used for emission. A 633 nm helium neon laser was used to excite the Alexa Fluor 647 signal and a 650–700 nm bandpass filter was used for emission. Fluorescence emission was observed, and a 100 × (numerical aperture, 1.4) oil immersion objective lens was used to visualize images. 200 nm *z*-step size stacks were obtained. The MATLAB program was used to analyze confocal images, which were obtained by noise filtering, edge detection and fluorescent signal extraction, so that different fluorescent signal channels could be extracted and quantified. Then, we obtained SGIV particles located on the cell membrane or in the cytoplasm. The co-localization of two different signals, e.g., SGIV and EcRab5c was also quantified by MATLAB program.

## Results

### EcRab5c Characterization

Based on EST sequences of EcRab5c from transcriptome data (Accession No. MT796123), the full-length open reading frame (ORF) of EcRab5c was obtained by PCR amplification. Sequence analysis revealed that EcRab5c encoded a 220 amino acid protein, with a high sequence similarity of 99% and 91% identity to *Anabas testudineus* and *Homo sapiens*, respectively. Amino acid alignments showed that EcRab5c contained five conserved domains, including G1–G5 (Supplementary Figure 1A). In mammalian systems, these G1–G5 regions are involved in GTP/GDP exchange, GTP hydrolysis and GTP-induced conformational changes (28). Phylogenetic analysis also revealed that EcRab5c was clustered to the fish branch, which is separate from amphibians, birds and mammals (Supplementary Figure 1B).

### Expression Patterns and Subcellular Localization of EcRab5c

**FIGURE 1:**
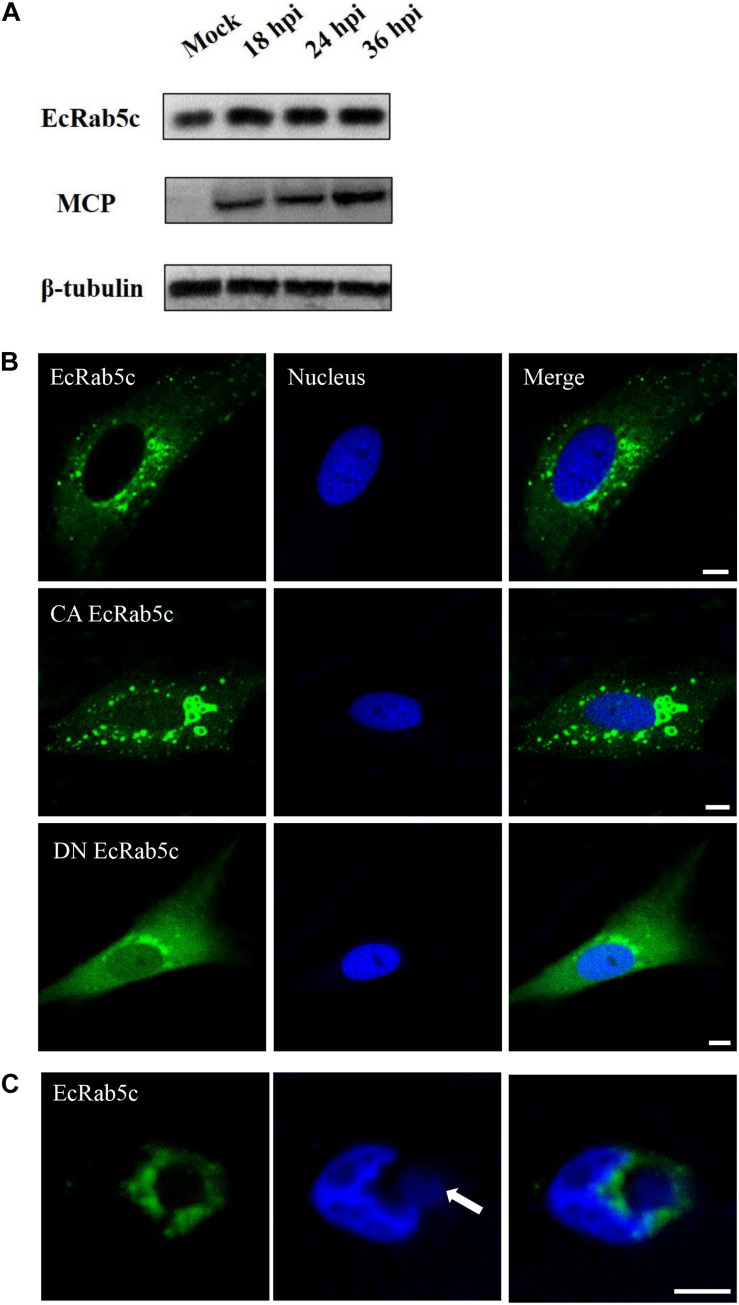
Expression patterns and subcellular distribution of EcRab5c. **(A)** EcRab5c and viral protein level were detected at different times post SGIV infection. GS cells were collected at 18, 24, and 36 hpi for Western blotting, and β-tubulin was used as the internal control. **(B)** Distribution pattern of EcRab5c, CA EcRab5c, and DN EcRab5c. GS cells were transfected with pEGFP-EcRab5c, pEGFP-CA EcRab5c, and pEGFP-DN EcRab5c, respectively. Scale bars are 5 μm. **(C)** EcRab5c located near the virus factory. GS cells transfected with pEGFP-EcRab5c were infected with SGIV and fixed at 24 h. The nucleus and virus factory were stained by Hoechst 33342. The white arrow shows the virus factory. Scale bars represent 10 μm.

To explore EcRab5c expression profiles after virus infection, the GS cells was infected with SGIV, and the expression of EcRab5c were examined by Western blotting. EcRab5c protein levels gradually increased with time post-SGIV infection, suggesting that EcRab5c may become activated and responds to SGIV infection (Figure 1A). In uninfected GS cells, EcRab5c were localized to typical punctate and vesicle-like structures in the cytoplasm (Figure 1B), similar to mammalian counterpart (29). It is reported that the switch between GTP- and GDP-bound formations is critical for the morphology and function of Rab5. In mammalian cells, the dominant-negative (DN) Rab5 mutant (Rab5; S34N), is defective for guanine-nucleotide binding, and maintains Rab5 in a continuously inactive state. In contrast, the constantly active (CA) Rab5 mutant (Rab5; Q79L) cannot hydrolyze GTP, causing the formation of enlarged EE. In this study, we constructed similar site-directed mutants of EcRab5c. As expected, CA EcRab5c (Q80L) overexpression in GS cells caused enlarged EE (Figure 1B), whereas, DN EcRab5c (S35N) overexpression led to protein dispersal and reduced vesicle structures, suggesting that continuous GTP/GDP cycle exchanges are critical for EcRab5c function in the grouper. We further investigated whether SGIV infection affected EcRab5c localization. As shown (Figure 1C), EcRab5c surrounded the virus factory at the late stage of virus infection, suggesting that EcRab5c was tightly associated with SGIV infection.

### EcRab5c Knockdown Inhibits SGIV Infection

**FIGURE 2:**
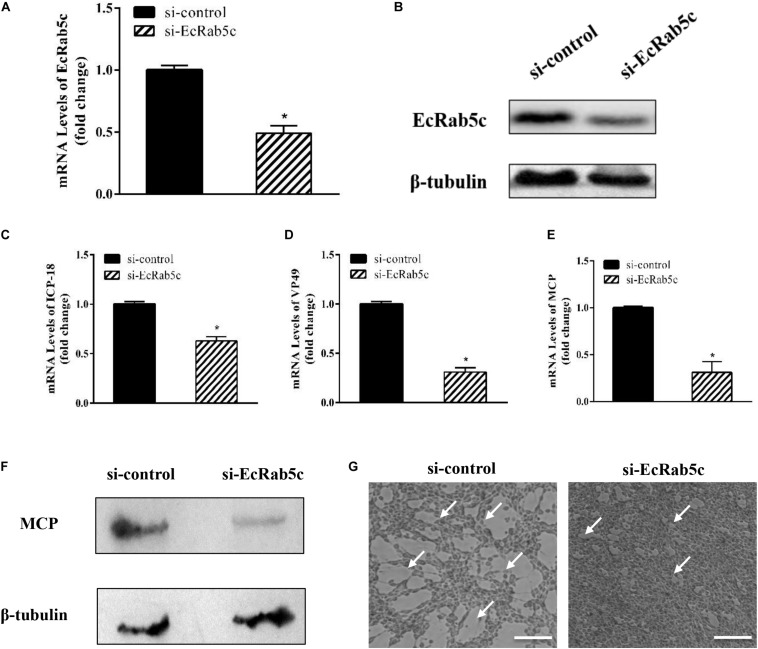
The si-EcRab5c significantly inhibited SGIV replication. **(A,B)** The knockdown efficiency of si-EcRab5c. GS cells were transfected with si-control or si-EcRab5c. After 36 h, the cells were harvested for qRT-PCR **(A)** and Western blotting **(B)**. β-tubulin was used as the internal control. **(C–E)** Knockdown of EcRab5c suppressed the expression of SGIV genes. GS cells were transfected with si-control or si-EcRab5c, incubated with SGIV at 12 h post transfection, and then collected at 24 hpi for further qRT-PCR analysis. Viral ICP-18 **(C)**, VP49 **(D)**, and MCP **(E)** were analyzed by qRT-PCR. The data were shown as fold change and indicated as the mean ± SEM (*n* = 3). Statistic differences are shown as *p < 0.05. **(F)** Knockdown of EcRab5c inhibited the protein expression of SGIV MCP. **(G)** Knockdown of EcRab5c reduced the CPE induced by SGIV. The arrows show the CPE. Scale bars represent 100 μm.

Given the importance of Rab5 in virus infection, we explored the influence of EcRab5c on SGIV infection by si-RNA interference. The efficient knockdown of EcRab5c was demonstrated by Western blotting; when si-EcRab5c was transiently transfected into GS cells, EcRab5c expression was significantly reduced (Figures 2A,B). Then, cells were infected with SGIV. As shown (Figure 2G), CPE were evident in si-EcRab5c cells, when compared with control cells. Consistently, when EcRab5c was knocked down, we observed that SGIV transcription levels of assorted viral genes, including ICP-18, VP49, and MCP, were significantly inhibited, i.e., reduced by approximately 50% (Figures 2C–E). Viral protein expression was also detected by Western blotting in EcRab5c-silenced cells, i.e., MCP expression was also reduced (Figure 2F). These data indicated that SGIV requires EcRab5c for successful infection.

### SGIV Cell Binding and Entry Depends on EcRab5c

**FIGURE 3:**
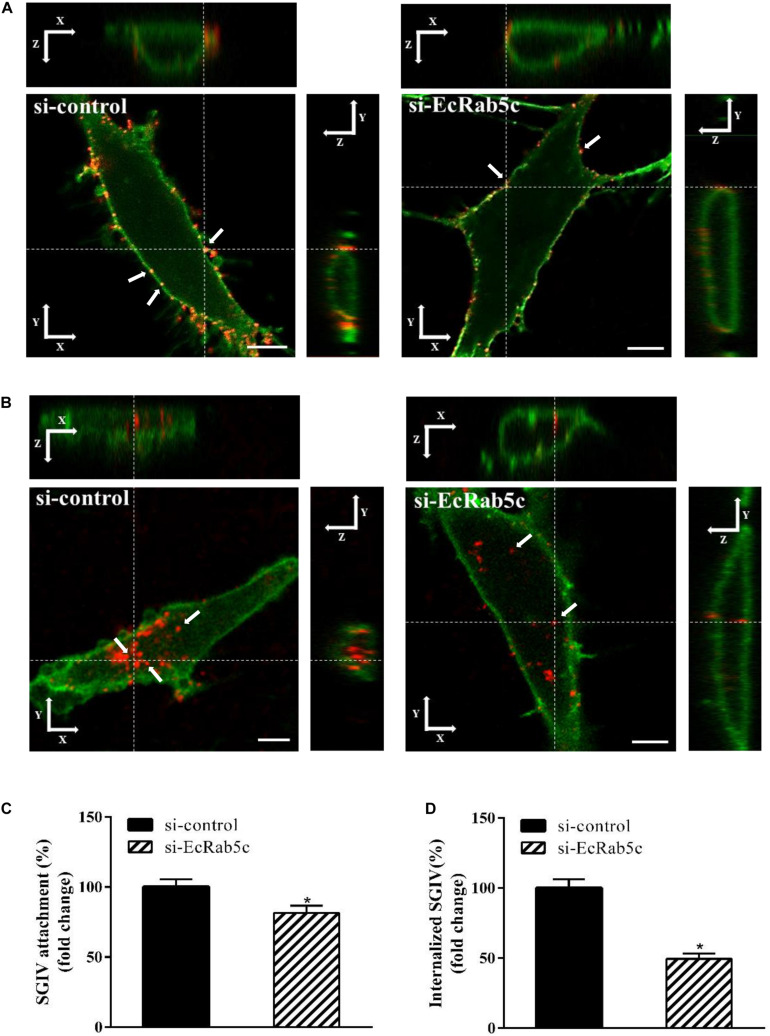
Knockdown of EcRab5c by siRNA affects the attachment and entry of SGIV. **(A)** Three-dimensional (3D) confocal images of SGIV attachment in si-control or si-EcRab5c transfected cells. GS cells were transfected with si-control or si-EcRab5c, incubated with Alexa-Fluor 647 labeled SGIV (red) at 4° C for 1 h, and then fixed with paraformaldehyde. The cells were stained with DiO to show the cell membrane (green), and observed by confocal microscope. White arrow indicated SGIV particles. Scale bars shown as 5 μm. **(B)** 3D confocal images of SGIV internalization in si-control or si-EcRab5c transfected cells. After transfection with si-control or si-EcRab5c, GS cells were incubated with Alex-Fluor 647 labeled SGIV (red) at 4° C for 20 min, and then the temperature was transferred to 28° C to initiate infection. The cells were fixed at 1 hpi. The samples were stained with DiO to indicate the cell boundaries (green). White arrow indicated SGIV particles. Scale bars represent 5 μm. **(C)** Quantification of SGIV particle binding on the cell membrane. More than 90 cells were randomly selected and analyzed by MATLAB program. The SGIV attachment was quantified as the percentage of virus particles binding on the cell membrane in si-EcRab5c transfected cells relative to that in si-control transfected cells. The value in control cells was arbitrarily set as 100%. The data are indicated as the means ± SEM. *p < 0.05. **(D)** Quantification of internalized SGIV particles. Over 90 cells were randomly selected and analyzed by MATLAB program. The SGIV uptake was quantified as the percentage of internalized virus particles in si-EcRab5c transfected cells relative to that in si-control transfected cells. The value in control cells was arbitrarily set as 100%. The data are indicated as the means ± SEM. *p < 0.05.

**FIGURE 4:**
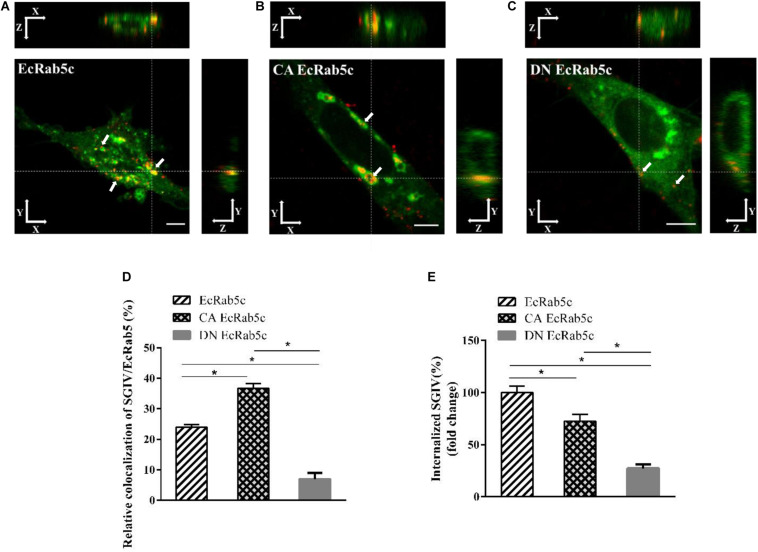
The mutant of EcRab5c affect the transportation of SGIV. **(A)** The 3D images of the colocalization of SGIV and EcRab5c. GS cells transfected with pEGFP-EcRab5c (Green) were incubated with Alex-Fluor 647 labeled SGIV (red) at 4° C for 20 min, transferred to 28° C, and then were fixed at 1hpi. White arrow indicated SGIV particles. Scale bars are 5 μm. **(B)** The 3D images of the colocalization of SGIV and CA EcRab5c. **(C)** The 3D images of the colocalization of SGIV and DN EcRab5c. **(D)** Quantification of SGIV particles colocalized with EcRab5c, CA EcRab5c and DN EcRab5c, respectively. Over 90 cells were randomly chose and analyzed by MATLAB program. The data are indicated as the mean ± SEM. *p < 0.05. **(E)** Quantification of internalized SGIV particles in EcRab5c, CA EcRab5c, or DN EcRab5c transfected cells. Over 90 cells were randomly chose and analyzed by MATLAB program. The data are indicated as the mean ± SEM. *p < 0.05.

Rab5 is known for its essential role in endocytosis, which includes endocytosis via caveolae or clathrin-coated vesicles, micropinocytosis and phagocytosis (1). We observed that EcRab5c is essential for SGIV infection, at early infection stages. Therefore, we explored the inhibitory effects of EcRab5c on virus attachment and entry using confocal imaging. In cells expressing si-EcRab5c, few SGIV particles appeared on the membrane, when compared with control cells (Figures 3A,C). These data implied that silencing Rab5 significantly impaired SGIV attachment. The influence of EcRab5c on SGIV entry was similarly characterized. When compared with the si-control, cells expressing si-EcRab5c showed significantly reduced SGIV signals, indicative of a block in SGIV entry (Figure 3B). Virus entry efficiency was quantified by determining fluorescently labeled SGIV particles in each cell. Silencing EcRab5c significantly decreased (50% of control value) SGIV uptake (Figure 3D). Consistent with data from knockdown experiments, DN, or CA EcRab5c overexpression strongly reduced internalized SGIV, by up to 73% and 28%, respectively (Figure 4E).

### EcRab5c Regulates SGIV Endocytic Transport

**FIGURE 5:**
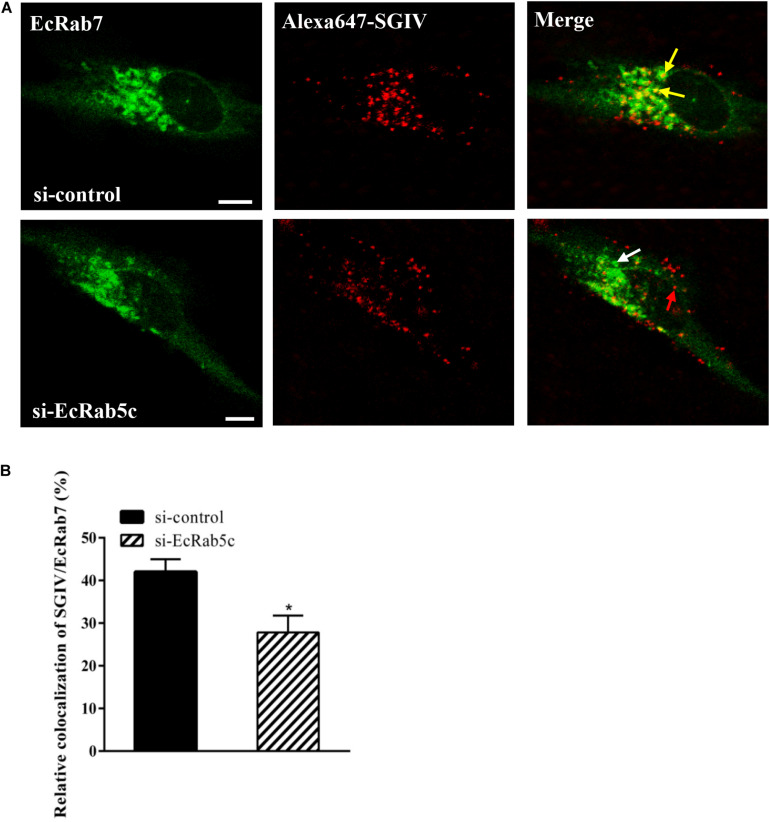
Knockdown of EcRab5c affect the colocalization of SGIV and EcRab7. **(A)** The confocal images of the colocalization between SGIV and EcRab7 in si-control or si-EcRab5c transfected cells. GS cells co-transfected with si-control/pEGFP-EcRab7 or si-EcRab5c/pEGFP-EcRab7 were incubated with Alex-Fluor 647 labeled SGIV at 4° C for 20 min, transferred to 28° C, and then were fixed at 1 hpi. Yellow arrows indicated the SGIV colocalized with EcRab7. White arrow indicated single EcRab7 signal. Red arrow indicated single SGIV signal. Scale bars represent 5 μm. **(B)** Quantification of SGIV particles colocalized with EcRab7 in **(A)**. Over 90 cells were randomly chose and analyzed by MATLAB program. The colocalization of SGIV/EcRab7 was quantified as the percentage of SGIV particles colocalized with EcRab7 to the total internalized virus particles. The colocalization of SGIV and EcRab7 in control cells was arbitrarily set as 100%. The data are indicated as the mean ± SEM. *p < 0.05.

Rab5 is a key regulator of endocytic trafficking (11). Hence, we further examined the impact of EcRab5c on the endocytic transport of SGIV. Our DN and CA Rab5 mutants provided powerful tools for study. As shown (Figures 4A,C,D), DN EcRab5c significantly inhibited SGIV particles located in Rab5-positive EE, indicative of a block in virus transport. On the contrary, CA EcRab5c significantly increased SGIV transport into large Rab5-positive vesicles (Figures 4A,B,D). Given that the conversion from Rab5 to Rab7 can be blocked by expressing CA Rab5, the virus particles delayed in CA EcRab5c-positive EE resulted from the failure of the switch between EE and LE. Upon internalization, SGIV was indeed transported into EE and then onto LE (24). The si-EcRab5c significantly blocked SGIV transport to the Rab7-positive LE (Figures 5A,B). These data indicate that EcRab5c plays an important role in the endocytic pathway of SGIV.

### Rab5c Positively Regulates the Autophagy Pathway

**FIGURE 6:**
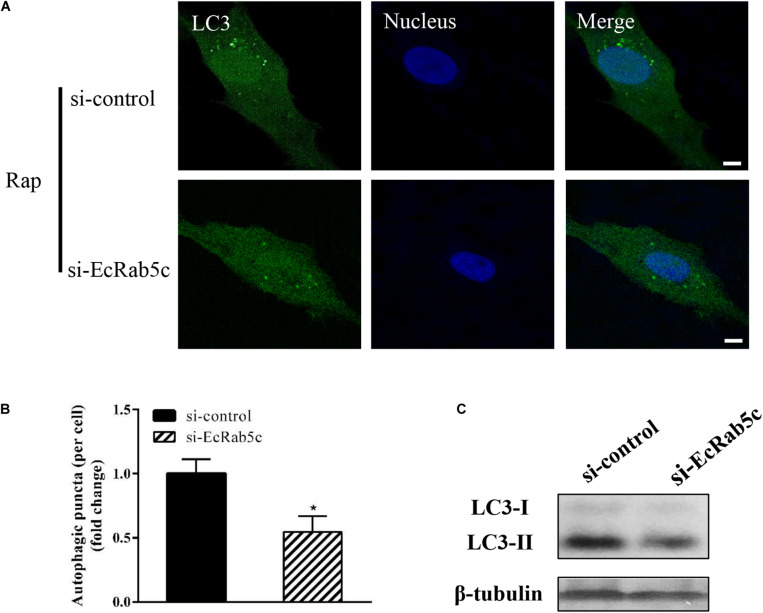
Knockdown of EcRab5c inhibited autophagy. **(A)** Expression of si-EcRab5c blocked autophagosome formation. GS cells were transfected with si-control/pEGFP-LC3 or si-EcRab5c/pEGFP-LC3, respectively, and then incubated with 1 μM Rap for 2 h. The nucleus was stained with Hoechst 33342. Scale bars are 5 μm. **(B)** Quantification of LC3 puncta/per cell in **(A)** was expressed as mean ± SEM, *n* = ∼90 cells of 3 independent experiments. *p < 0.05. **(C)** Expression of si-EcRab5c reduced the level of LC3-II by Western blotting.

**FIGURE 7:**
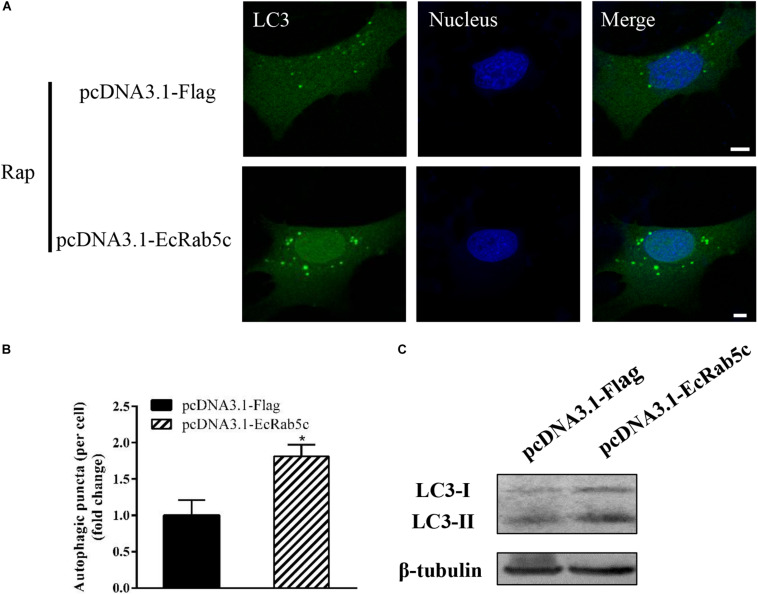
Overexpression of EcRab5c promoted autophagy. **(A)** Overexpression of EcRab5c enhanced autophagosome formation. GS cells were transfected with pcDNA3.1-Flag/pEGFP-LC3 or pcDNA3.1-EcRab5c/pEGFP-LC3, respectively, and then incubated with 1 μM Rap for 2 h. The nucleus was stained with Hoechst 33342. Scale bars are 5 μm. **(B)** Quantification of LC3 puncta/per cell in **(A)** was expressed as mean ± SEM, *n* = ∼90 cells of 3 independent experiments. *p < 0.05. **(C)** Overexpression of EcRab5c increase the level of LC3-II by Western blotting.

Autophagy is an ancient and highly conserved intracellular degradation process, and Rab5 is reported to participate in autophagosome formation (30). Accordingly, we speculated if EcRab5c was also involved in the autophagy process. The microtubule-associated protein light chain 3 (LC3) is commonly used as a specific marker to identify autophagosome formation (31). Hence, we used EGFP- tagged LC3 to indicate autophagosomes by fluorescent LC3 puncta (32). We first tested whether EcRab5c knockdown affected autophagosome formation. After Rap treatment (an autophagic inducer that inhibits the mammalian target of Rap pathway), LC3 patterns in cells co-transfected with si-control and pEGFP-LC3 showed punctate morphology, characteristic of autophagosome structures (Figure 6A). However, in cells co-transfected with si-EcRab5c and pEGFP-LC3, little LC3 puncta were observed, and the numbers of LC3 puncta were significantly decreased, suggesting that EcRab5c silencing impaired autophagosome formation (Figure 6B). LC3 convert from LC3-I, the cytosolic form, to LC3-II, the lipidated and autophagosome-associated form, and LC3-II is widely used as a hall marker of autophagy (33). To further verify the role of EcRab5c in autophagy, we performed Western blotting to monitor LC3-II levels. We observed a significant decrease in LC3-II protein levels, and the ratio of LC3-II to LC3-I was much lower in si-EcRab5 transfected cells, when compared to cells transfected with si-control (Figure 6C). However, EcRab5c overexpression significantly enhanced autophagy (Figures 7A–C). These data suggested that EcRab5c positively regulated autophagy.

### si-Rab5c Impairs IFN Immune and Inflammatory Responses

**FIGURE 8:**
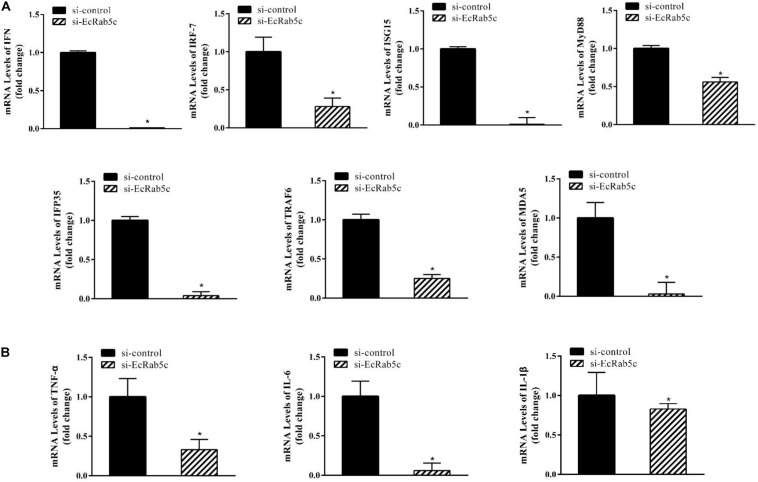
Knockdown of EcRab5c inhibited IFN-signaling molecules **(A)** including IFN, IRF-7, ISG15, MyD88, IFP35, TRAF6, MDA5, and pro-inflammatory cytokines **(B)** including TNF-α, IL-6, and IL-1β. GS cells were transfected with si-control or si-EcRab5c. After 36 h, the cells were collected for qRT-PCR (*n* = 3, mean ± SEM). *p < 0.05.

**FIGURE 9:**
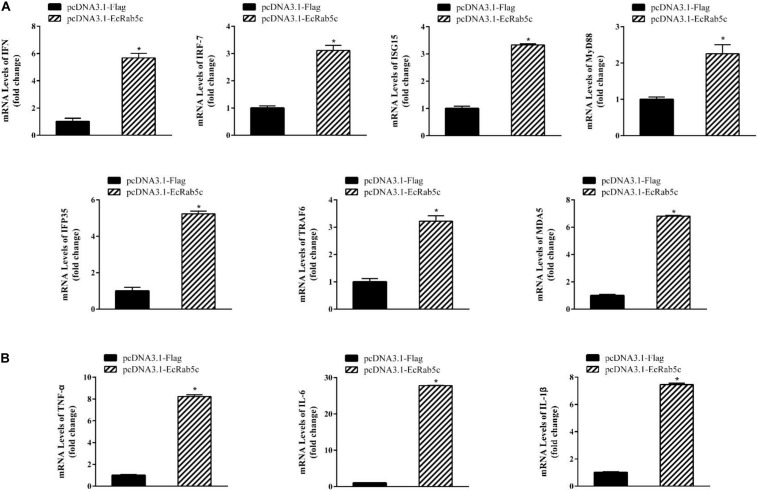
Overexpression of EcRab5 positively regulated IFN-signaling molecules **(A)** including IFN, IRF-7, ISG15, MyD88, IFP35, TRAF6, MDA5, and pro-inflammatory cytokines **(B)** including TNF-α, IL-6, and IL-1β. GS cells were transfected with pcDNA3.1-Flag or pcDNA3.1-EcRab5c. After 24 h, the cells were collected for qRT-PCR (*n* = 3, mean ± SEM). *p < 0.05.

It is reported that Rab GTPases also regulate several immune response components (1). To clarify the function of Rab5c in host IFN immune and inflammatory responses, we examined the expression of IFN signaling molecules and pro-inflammatory cytokines by qRT-PCR. When compared with controls, Rab5c knockdown significantly reduced the expression of several IFN-related genes, including IFN regulatory factor (IRF)-7, IFN-induced protein (IFP)-35, IFN-stimulated gene (ISG)-15, myeloid differentiation primary response gene (MyD)-88, TNF receptor associated factor (TRAF)-6, melanoma differentiation-associated gene (MDA)-5, and type I IFN (Figure 8A). However, these IFN-related genes were significantly increased in EcRab5c-overexpressing cells (Figure 9A). In addition, tumor necrosis factor (TNF)-α, IL-6, and IL-1β gene expression was significantly decreased by si-EcRab5c (Figure 8B). However, these genes were significantly up-regulated in EcRab5c-overexpressing cells (Figure 9B). Given that Rab proteins play important roles in cellular signaling, it is proposed that EcRab5c is a potential regulator in IFN immune and inflammatory responses.

## Discussion

Rab GTPases are highly conserved molecules across most eukaryotic cell systems, even in mammals and yeast (1). The main endosomal GTPase is Rab5, localizing to endosome membranes and regulating endocytic pathways (34). Rab5 has three isoforms, Rab5a, -b, and -c, with some overlapping roles, but distinct functions (26). While Rab5a has been extensively studied, Rab5c has gained very little attention. In this study, we cloned a novel Rab5c from *E. coioides*, and explored its specific roles in SGIV infection and cellular immune responses.

EcRab5c showed high amino acid sequence identity with homologs from other species, such as *H. sapiens*, suggesting an evolutionary conserved role in endosomal biogenesis. Moreover, by introducing specific mutations at conserved residues, CA and DN EcRab5c were constructed to maintain Rab5c in continuously GTP- or GDP-bound state, respectively. This led to enlarged or reduced vesicles, suggesting that GTP/GDP cycles are also critical for EcRab5c, suggesting EcRab5c may have similar activation mechanisms and functions as its mammalian homologs. Furthermore, EcRab5c was up-regulated after SGIV infection. Other parasitic infections can also affect Rab5 expression. After catfish were infected with *Edwardsiella ictaluri*, Rab5c was up-regulated in the head and trunk kidney, suggesting a potential role in acute immune responses (35). Rab5 transcription was significantly increased in the spleen of the large yellow croaker after an immune challenge, and a *Vibrio parahaemolyticus* infection (36).

Many studies, including those on IV, hepatitis B virus, bovine ephemeral fever virus and classical swine fever virus, have shown that Rab5 is tightly associated with virus infection (37–40). In our study, EcRab5c is also a key factor in SGIV infection. EcRab5c knockdown significantly inhibited SGIV infection. These inhibitory effects may have resulted from the dual role of EcRab5c. Specifically, si-EcRab5c RNA reduced SGIV attachment, suggesting that EcRab5c inhibition may impair receptor recycling, thereby reducing virus receptors from cell surfaces, and simultaneously decreasing virus attachment, entry and infection. It has been reported that Rab5 activation positively regulates EGFR internalization (41, 42). Unfortunately, the virus receptor used by SGIV has not been identified. So we could not confirm that Rab5 affect SGIV attachment or entry by its effect on virus receptor. But our observations cannot exclude the possibility that EcRab5c regulates SGIV receptors on cell membranes. In addition to receptor internalization, Rab5 plays roles in CME and macropinocytosis, which are entry routes for SGIV in GS cells (24). Our results showed that EcRab5c knockdown and CA and DN EcRab5c both significantly blocked SGIV entry, suggesting that EcRab5c regulates endocytosis during virus entry. Other viruses, such as AdV and HCV, also depends on Rab5 for uptake (15, 17).

Furthermore, apart from its role in endocytosis, Rab5 also participates in endocytic trafficking, including EE formation and maturation, and is involved in the endocytic transport of many viruses, such as IV, foot-and-mouth disease virus, and human immunodeficiency virus (38, 41, 43). EcRab5c disruption significantly affected SGIV trafficking through the endocytic pathway. DN EcRab5c impaired SGIV access to the EE, while CA EcRab5c trapped SGIV in large Rab5-positive vesicles. Moreover, si-EcRab5c significantly blocked SGIV transport to the LE. Hence, except for entry, EcRab5c is required for the delivery of SGIV to EE, LE, and even lysosomes.

In addition, Rab5 also participates in the RNA replication of HCV and forming replication complexes (16, 17). HSV1 envelopment also requires Rab5 (18). However, it is not clear whether EcRab5c affects viral gene replication, transcription or SGIV assembly. Although si-EcRab5c significantly inhibited viral gene transcription as detected by qRT-PCR, this inhibition could be caused by decreased SGIV uptake. Additionally, the fact that EcRab5c surrounds the SGIV virus factory, suggests that EcRab5c may play a role in the late stages of virus infection (Figure 1C).

A growing number of studies have revealed that Rab5 is involved in autophagy regulation (12). Rab5 appears to regulate the mammalian target of Rap (mTOR) kinase activity, and is associated with Beclin l and Vps34 to form a complex, essential for autophagosome formation (30, 44, 45). Furthermore, Rab5 inhibition resulted in the down-regulation of autophagosome formation, by reducing Atg5-Atg12 conjugation with Vps34 (46). Vps34, a class III phosphoinositide 3-kinase (PI3K), with a role in autophagy initiation, has been identified as a Rab5 effector (47, 48). On the contrary, Rab5 inhibition activates the autophagy in *Caenorhabditis elegans* (49). Thus, to further explore whether Rab5c is involved in fish autophagy, EcRab5c was knocked down by si-RNA, resulting in a significant decrease in LC3-II expression. Imaging and quantification data showed that EcRab5c knockdown significantly inhibited autophagosome formation. Based on these observations, it is important to note that EcRab5c participates in autophagy pathways, therefore Rab5c inhibition may interrupt autophagy and affect cellular catabolic processes in other species. This is the first time to unveil the role of Rab5c in autophagy.

Apart from virus infection, Rab5 has been linked to IFN immune and inflammatory responses (50). EcRab5c also demonstrated such associations. The mRNA levels of the IFN signaling molecules, IFN, IRF-7, ISG15, MyD88, IFP35, TRAF6, MDA5, and pro-inflammatory factors, TNF-α, IL-6, and IL-1β were all significantly decreased in EcRab5c silenced cells, but increased in EcRab5c overexpressing cells. It is inferred that EcRab5c may affect receptor uptake, transport and then signal transduction. For example, Rab5 disruption may modulate the activity of at least two independent the signal transducer and activator of transcription (STAT) molecules (50, 58). In the *Larimichthys crocea*, Rab5 overexpression up-regulated TNF-α and IL-6 expression, whereas cytokines also affected Rab5 expression; in immune cells, IL-4 and 6 up-regulated Rab5 expression, and the synthesis of Rab5a was also increased after IFN-γ stimulation in mononuclear cell (36, 51–53). In addition, pro-inflammatory cytokines were also associated with autophagy activation (54). IL-1β activated autophagy to control pathogens infection, such as *M. tuberculosis* (55–57). Therefore, EcRab5c inhibitory effects on autophagy may elicit several effects; EcRab5c disruption directly reduces autophagosome formation, or it may also decrease autophagy levels by down-regulating inflammatory cytokines. Whether inflammatory cytokines regulate autophagy pathways in fish still requires further study.

As a crucial mediator in regulating intracellular trafficking pathways, Rab5 are required for many viruses infection and also plays an important role in the immune system (12, 38, 41, 43). In this study, EcRab5c could play a bi-function role in SGIV infection and regulating the cellular immune response. On the one hand, the disruption of EcRab5c significantly inhibits SGIV infection. On the other hand, EcRab5c is critical regulator of host immunity.

Innate immune response is the first defense when virus infected host cells. Generally, disruption innate immune response would promote virus infection. Inhibition autophagy indeed promotes SGIV infection (unpublished data). EcRab5c knockdown resulted in the inhibition of autophagy, IFN and inflammatory responses, which was supposed to benefit SGIV infection. However, EcRab5c knockdown significantly inhibited SGIV infection, suggesting that SGIV must rely on EcRab5c for successful infection. EcRab5c mainly affect SGIV infection by regulating the endocytic pathway of SGIV.

In conclusion, we comprehensively explored the roles of EcRab5c in SGIV infection and cellular immunity. EcRab5c was cloned from the orange-spotted grouper, and up-regulated after SGIV infection. Knockdown or disruption of EcRab5c significantly inhibited SGIV infection, including virus binding, entry, and transport. Equally, EcRab5c positively regulated autophagy, IFN signaling molecules, and pro-inflammatory factors. These results suggest a complex roles for EcRab5c in viral infection and host immunity.

## Data Availability Statement

All datasets presented in this study are included in the article/Supplementary Material.

## Author Contributions

LW and SWa conceived the experiments, wrote the manuscript, analyzed the data, and contributed materials/analysis tools. CL, XZ, MY, and SWe contributed reagents, materials, and analysis tools for the work. QQ supervised the study, reviewed drafts of the manuscript, and provided final approval of the version to be published. The other authors participated in the data analyses. All authors discussed the results and commented on the manuscript.

## Conflict of Interest

The authors declare that the research was conducted in the absence of any commercial or financial relationships that could be construed as a potential conflict of interest.

## Footnotes

1 http://www.ncbi.nlm.nih.gov/blast

2 http://smart.embl-heidelberg.de/

3 http://www.ebi.ac.uk/clustalW/
