# Supplementary material for: The Small GTPase Rab5c Exerts Bi-Function in Singapore Grouper Iridovirus Infections and Cellular Responses in the Grouper, Epinephelus coioides
Source: Front Immunol. 2020 Sep 2;11:2133. doi: 10.3389/fimmu.2020.02133 (PMC7495150; doi:10.3389/fimmu.2020.02133)
Supplement: Supplementary file 1 [file Data_Sheet_1.pdf]

Supplementary Material

The Small GTPase Rab5c Exerts Bi-function in Singapore Grouper Iridovirus Infections and Cellular Responses in the Grouper, *Epinephelus coioides*

Liqun Wang[1], Chen Li[2], Xinyue Zhang[1], Min Yang[1], Shina Wei[1], Youhua Huang[1], Qiwei Qin[1,3,4], Shaowen Wang[1,3*]**

[1] Joint Laboratory of Guangdong Province and Hong Kong Region on Marine Bioresource Conservation and Exploitation, College of Marine Sciences, South China Agricultural University, Guangzhou, China

[2] College of Fisheries, Henan Normal University, Xinxiang, China

[3] Guangdong Laboratory for Lingnan Modern Agriculture, Guangzhou, China

[4] Laboratory for Marine Biology and Biotechnology, Qingdao National Laboratory for Marine Science and Technology, Qingdao, China

*** Correspondence:**
Shaowen Wang

wangsw@scau.edu.cn

****Co-correspondence:**

Qiwei Qin

qinqw@scau.edu.cn

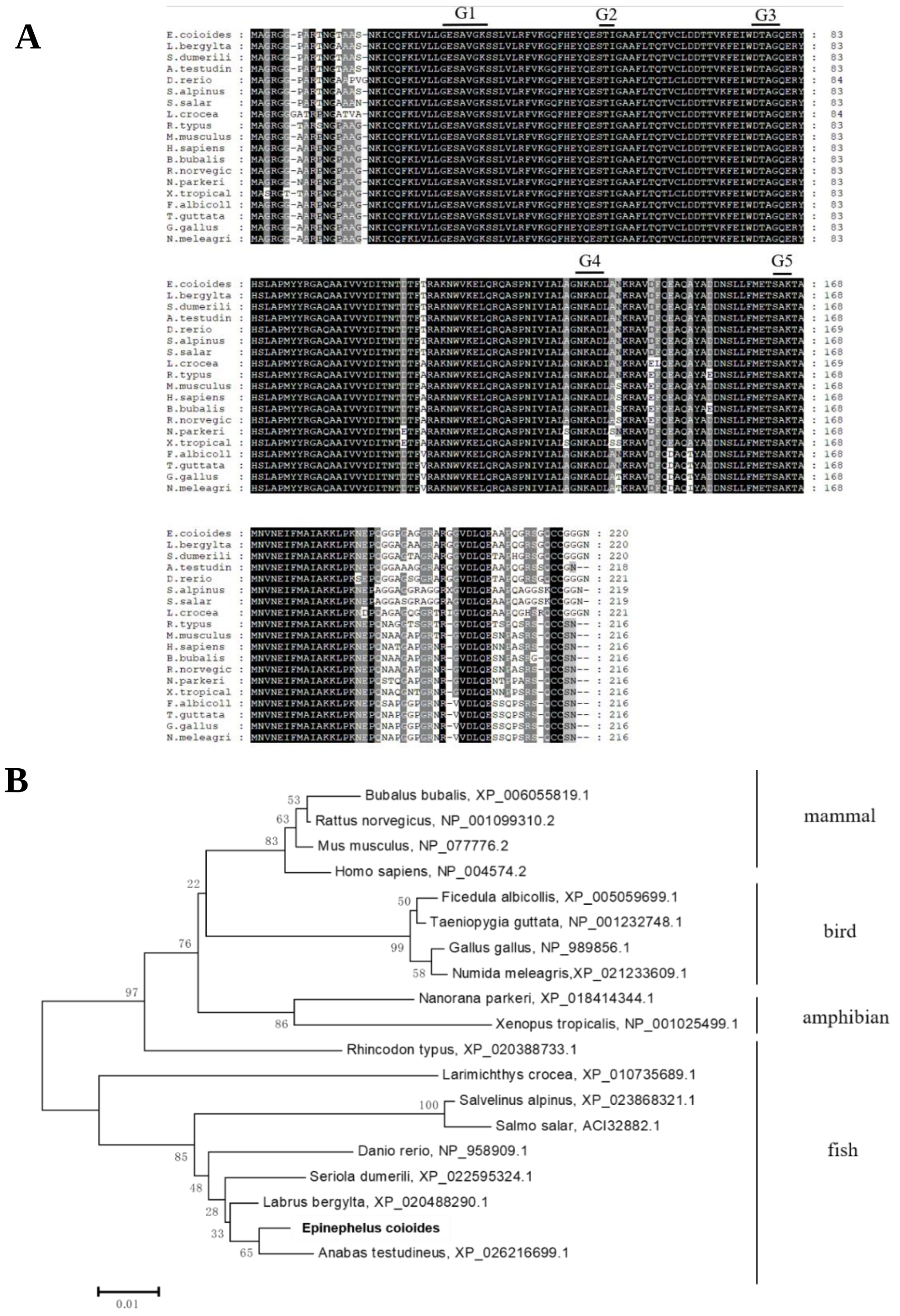

Supplementary Figure 1. (A) Amino acid sequence alignments of Rab5c from different species. The conserved domains G1, G2, G3, G4 and G5 were marked above the sequences. (B) Phylogenetic tree of Rab5c proteins. Phylogenetic tree was constructed by the neighbor-joining method using MEGA 6.0 software. The accession number of selected Rab5c sequences were listed on the right of species name.

Supplementary Table 1 Sequence of primers and si-EcRab5c used in this study

Name	Sequence (5′-3′)
RT- EcRab5c-F	GGACAGGAACGGTATCACAG
RT- EcRab5c-R	CTCCTTCACCCAGTTCTTCG
RT- Actin-F	TACGAGCTGCCTGACGGACA
RT- Actin-R	GGCTGTGATCTCCTTCTGCA
RT- ICP18-F	ATCGGATCTACGTGGTTGG
RT- ICP18-R	CCGTCGTCGGTGTCTATTC
RT- VP49-F	CCCGCAATGAACTCGCCAAAACT
RT- VP49-R	CCGTGACGTACTGCCAAGCCTGA
RT- MCP-F	GCACGCTTCTCTCACCTTCA
RT- MCP-R	AACGGCAACGGGAGCACTA
EcRab5c-ORF-F	ATGGCAGGGCGAGGCGGA
EcRab5c-ORF-R	GTTCCCGCCCCCACAGC
pEGFP-EcRab5c-F	GGGGTACCATGGCAGGGCGA
pEGFP-EcRab5c-R	CGGGATCCGTTCCCGCCCCCACAGC
pcDNA3.1-EcRab5c-F	GGGGTACCGAATGGCAGGGCGAGGCGGA
pcDNA3.1-EcRab5c-R	CGGAATTCGTTCCCGCCCCCACAGC
RT- IFN-F	ACTGTGTCCTTCCCGAATCATCTGT
RT- IFN-R	CAGCCTGCCTGCTTACAACACTGAGAA
RT- IRF-7-F	CAACACCGGATACAACCAAG
RT- IRF-7-R	GTTCTCAACTGCTACATAGGGC
RT- ISG15-F	CCTATGACATCAAAGCTGACGAGAC
RT- ISG15-R	GTGCTGTTGGCAGTGACGTTGTAGT
RT- MyD88-F	AGCTGGAGCAGACGGAGTG
RT- MyD88-R	GAGGCTGAGAGCAAACTTGGTC
RT- IFP35-F	TTCAGATGAGGAGTTCTCTCTTGTG
RT- IFP35-R	TCATATCGGTGCTCGTCTACTTTCA
RT- TRAF6-F	CCCTATCTGCCTTATGGCTTTGA
RT- TRAF6-R	ACAGCGGACAGTTAGCGAGAGTAT
RT- MDA5-F	ACCTGGCTCTCAGAATTACGAACA
RT- MDA5-R	TCTGCTCCTGGTGGTATTCGTTC
RT- TNF-α-F	GTGTCCTGCTGTTTGCTTGGTA
RT- TNF-α-R	CAGTGTCCGACTTGATTAGTGCTT
RT- IL-6-F	GGTTGGTCCAAGGTGTGCTTA
RT- IL-6-R	CTGGGATTGTCGAGGTCCTT
RT- IL-1β-F	AACCTCATCATCGCCACACA
RT- IL-1β-R	AGTTGCCTCACAACCGAACAC
pEGFP-EcRab5c-Q80L-F	ACTGCAGGACTAGAACGGTATCACAGCTTGGCACCCATGTA
pEGFP-EcRab5c-Q80L-R	ATACCGTTCTAGTCCTGCAGTGTCCCAGATCTCAAACTTG
pEGFP-EcRab5c-S35N-F	GTGGGGAAGAACAGCTTGGTGCTGCGCTTTGTCAAAGGCCA
pEGFP-EcRab5c-S35N-R	CACCAAGCTGTTCTTCCCCACTGCTGATTCCCCCAACAGC
The labeled si-EcRab5c	AGACUGCUAUGAAUGUCAAUGAGAU-cy3
